# Dual regulation of transcription factor binding by tup11/12-mediated destabilization and DNA loop-mediated stabilization ensures stress-specific gene activation in fission yeast

**DOI:** 10.1093/nar/gkag847

**Published:** 2026-09-07

**Authors:** Yusuke Tsuruta, Hirotaka Inoue, Kenta Hagiwara, Charles S Hoffman, Kouji Hirota

**Affiliations:** Department of Chemistry, Graduate School of Science, Tokyo Metropolitan University, Minamiosawa 1-1, Hachioji-shi, Tokyo 192-0397, Japan; Department of Chemistry, Graduate School of Science, Tokyo Metropolitan University, Minamiosawa 1-1, Hachioji-shi, Tokyo 192-0397, Japan; Department of Chemistry, Graduate School of Science, Tokyo Metropolitan University, Minamiosawa 1-1, Hachioji-shi, Tokyo 192-0397, Japan; Biology Department, Boston College, Chestnut Hill, MA 02467, United States; Department of Chemistry, Graduate School of Science, Tokyo Metropolitan University, Minamiosawa 1-1, Hachioji-shi, Tokyo 192-0397, Japan

## Abstract

An appropriate transcriptional response to external stresses is crucial for all organisms. Gene expression is regulated by transcription factors (TFs) binding to specific DNA *cis-*elements. However, how individual TFs achieve stress-specific binding remains elusive. Here we examine the molecular basis of the stress-specific transcriptional response of the *Schizosaccharomyces pombe fbp1* gene. The *fbp1* gene is activated upon glucose starvation, with transcriptional co-repressors Tup11 and Tup12 playing pivotal roles in maintaining stress-specificity. In the absence of Tup11 and Tup12, nonspecific activation of *fbp1* transcription occurs, which requires the TFs Atf1 and Rst2—both essential for *fbp1* induction. Moreover, this aberrant *fbp1* activation is diminished by the loss of Php5, a factor indispensable for DNA-loop formation between Atf1- and Rst2-binding sites. The defective nonspecific transcription in the *tup11∆/tup12∆/php5∆* mutant is restored when Atf1- and Rst2-binding sites are artificially positioned in proximity to each other, indicating that reciprocal stabilization between these TFs facilitates their binding. These findings demonstrate that *fbp1* achieves stress-specific transcriptional activation through the counteractive regulation of TF-binding: Tup11/12-mediated destabilization and DNA loop-mediated stabilization. The synergistic reduction in stress tolerance observed in the *tup11∆/tup12∆/php5∆* mutant further suggests that this dual regulatory mechanism is crucial for cellular adaptation to environmental stresses.

## Graphical Abstract

**Graphical Abstract:**
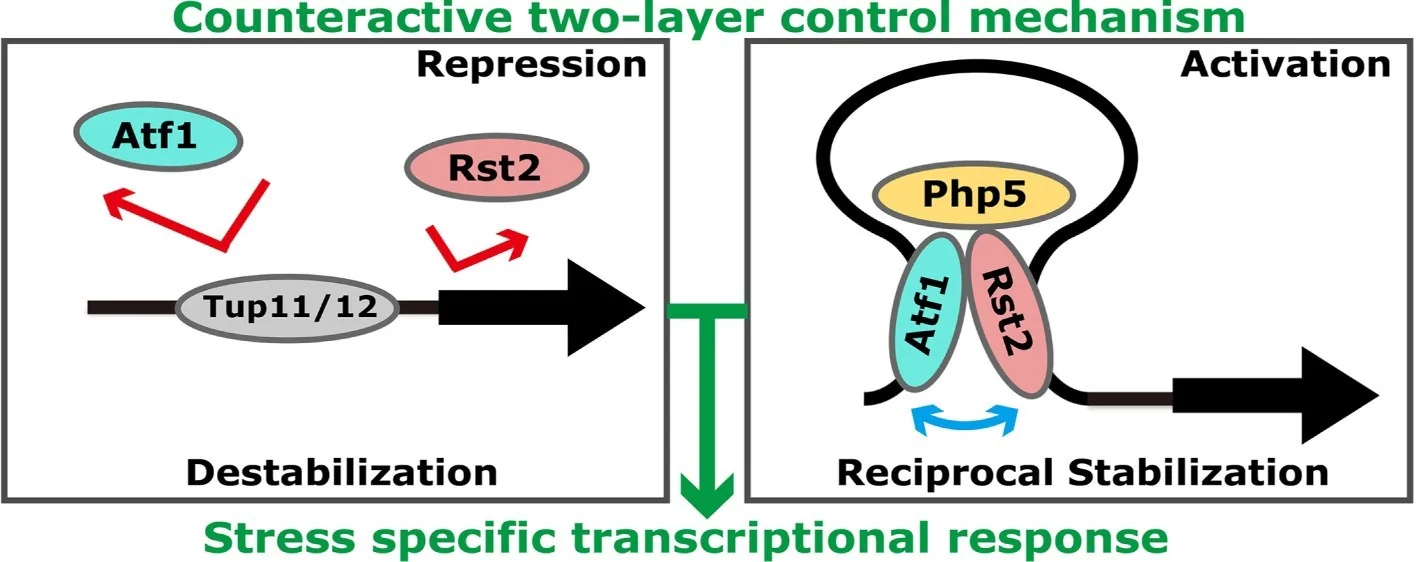

## Introduction

Cells have evolved diverse signaling systems to respond to fluctuations in the external environment. These signaling pathways transmit extracellular cues to activate specific transcription factors (TFs), which in turn, elicit appropriate transcriptional programs that enable cells to withstand environmental stresses [1, 2]. However, the molecular mechanisms by which cells decode different stresses—such as heat stress, nutrient deprivation, and DNA damage—have not been fully elucidated.

In *Schizosaccharomyces pombe*, the *fbp1* gene, encoding fructose-1,6-bisphosphatase, serves as an established model for stress-responsive transcription. This gene is stringently repressed under glucose-rich conditions and selectively activated during glucose starvation [3, 4]. Upon glucose depletion, two independent signaling pathways transmit the stress signal. One is the stress-activated protein kinase (SAPK) pathway, in which the SAPK Spc1/Sty1 is activated by phosphorylation [5, 6]. The other is the protein kinase A (PKA) pathway, which senses glucose availability through intracellular concentration of cyclic AMP (cAMP) levels [4, 7, 8]. High cAMP levels promote the dissociation of Cgs1, the regulatory subunit of PKA, from the Pka1 catalytic subunit thereby activating PKA. Conversely, glucose starvation depletes cAMP, leading to inactivation of PKA [9]. Under these conditions, two TFs—Atf1, a CREB/ATF family TF, and Rst2, zinc-finger TF—are phosphorylated and activated under the regulation of SAPK and PKA pathways, respectively [10–16]. Both factors are indispensable for *fbp1* transcription and bind to distinct regulatory elements named upstream activation sequences 1 and 2 (UAS1 and UAS2), respectively, in the *fbp1* upstream region [17]. UAS1 contains a cAMP-responsive element (CRE), whereas UAS2 harbors a CT-rich stress response element (STRE). The transcriptional co-repressors Tup11 and Tup12 (collectively Tup11/12) are required for the stringent repression of *fbp1* under glucose-rich conditions [18, 19]. Intriguingly, in *tup11∆/tup12∆* cells, *fbp1* is aberrantly induced under other stresses such as nitrogen starvation stress, suggesting that Tup11/12 act not merely as simple repressors but as transcriptional regulators ensuring stress-specificity [20].

The binding of Atf1 and Rst2 to UAS1 and UAS2 is regulated by two distinct mechanisms. First, the chromatin configuration around these *cis-*elements is modulated by noncoding RNA transcription. Under glucose-rich conditions, the chromatin condensation around UAS1 and UAS2 suppresses the binding of these TFs [18]. During glucose starvation, a series of long noncoding RNAs termed metabolic stress-induced long noncoding RNAs (mlonRNAs) are transcribed in a stepwise manner from upstream to downstream of UAS1 [21]. These stepwise mlonRNA initiations progressively convert chromatin into an open configuration, thereby permitting TF-binding [21] (Supplementary Fig. S1A). Secondly, the spatial positioning of Rst2 is regulated through DNA-loop formation between UAS1 and UAS2 [22]. Following chromatin relaxation induced by mlonRNA transcription, Atf1 and Rst2 initially bind to UAS1 and a nearby upstream site respectively, subsequently stabilizing reciprocally at UAS1 region [23] (Supplementary Fig. S1B). This reciprocal stabilization counteracts Tup11/12-mediated repression of TF binding at the UAS1 region [23]. Thereafter, the CBF/NF-Y-type TF Php5 promotes loop formation between UAS 1 and UAS2 [22], bringing the two regions into close proximity. This process antagonizes Tup11/12-mediated repression of Rst2 biding at UAS2 and enables Rst2 to migrate from upstream of UAS1 to UAS2, ultimately activating *fbp1* messenger RNA (mRNA) transcription [22] (Supplementary Fig. S1B). Nevertheless, the precise mechanism by which Tup11/12 safeguards the stress-specificity and the manner in which Atf1, Rst2, and Php5 cooperate under specific-stress condition remain to be elucidated.

Increasing evidence suggests that TF occupancy is not solely determined by DNA-binding specificity, but is also shaped by cooperative interactions among TFs [23, 24], chromatin architecture [25], and higher-order assemblies such as transcriptional condensates [26–28]. Multivalent interactions among regulatory factors can stabilize promoter occupancy by increasing local factor concentration and residence time, thereby promoting productive transcriptional activation. In contrast to these multiple layers of TF stabilizing mechanisms, Tup family proteins destabilize TF binding by maintaining promoter nucleosome positioning and occupancy [29]. However, mechanisms of how Tup proteins safeguard the stress-specificity and how this regulatory mechanism relates to other numerous regulatory layers on transcription have been elusive.

In this study, we investigated the mechanism of how Tup11/Tup12 and three TFs establish the appropriate *fbp1* transcriptional response by analyzing factors responsible for the aberrant *fbp1* activation in response to nitrogen starvation in *tup11∆/tup12∆* cells. We found that this aberrant *fbp1* induction requires Atf1 and Rst2, and that Php5-mediated DNA-loop formation is indispensable for this nonspecific *fbp1* induction in the absence of Tup11/12. Remarkably, the defective *fbp1* activation in *tup11∆/tup12∆/php5∆* cells is restored when UAS1 and UAS2 are artificially placed in proximity to each other. These findings suggest that *fbp1* achieves stress-specific transcription through counteractive regulation: destabilization of TF-binding by Tup11/12 and TF stabilization by DNA-loop-mediated proximity. Transcriptome analyses further revealed that this dual regulatory mechanism also plays a role in the regulation of other genes in fission yeast genome. Moreover, the concurrent loss of Tup11/12 and Php5 leads to a synergistic reduction in cellular survival to a wide variety of stresses. These findings indicate that this dual regulatory mechanism plays a critical role in cellular adaptation to environmental stresses.

## Materials and methods

### Fission-yeast strains, genetic methodology, and cell culture

The fission yeast strains used in this study are listed in Table 1. YER medium (yeast extract containing 6% glucose) and YED medium (yeast extract containing 0.1% glucose and 3% glycerol) were used for glucose-rich and starvation, respectively [18]. For nitrogen starvation, MM − N medium (0.3% KH phthalate, 0.22% Na_2_HPO_4_, 1% glucose, 2% salt stock [5.53% Mg_2_Cl_2_∙6H_2_O, 0.0735% CaCl_2_∙2H_2_O, 5.0% KCl, 0.2% Na_2_SO4], 0.1% 3-vitamin stock [0.1% pantothenate Ca, 1% nicotinic acid, 1% inositol], 0.1% biotin stock [0.001% biotin], and 0.1% trace element [0.05% H3BO3, 0.53% MnSO_4_∙7H_2_O, 0.2% FeCl_3_∙6H_2_O, 1% (NH_4_)_6_Mo_7_O_24_∙4H_2_O, 0.1% KI, 0.04% CuSO_4_∙5H_2_O]) supplemented with required amino acids was used as previously reported [30]. The transformation was performed using the lithium-acetate method as previously described [31].

**Table 1.** Fission yeast strains used in this study

Strain number	Genotype	References
SPH1	*h^−^ leu1-32*	[32]
SPH2	*h + ura4-D18*	[33]
SPH13	*h^−^ ade6-M26 leu1-32 ura4-D18 tup11::ura4 tup12::ura4*	[17, 18]
SPH19	*h^−^ ade6-M26 leu1-32 rst2::kanMX6*	[20]
SPH20	*h^−^ ade6-M26 rst2-3Flag<<kanMX6 leu1-32*	[23]
SPH117	*h^+^ ura4-D18 php5::kanMX6*	[22]
SPH125	*h^+^ ade6-M26 rst2-3flag<<kanMX6 leu1-32 ura4-D18*	[23]
SPH141	*h^−^ ade6-M26 leu1-32 ura4-D18 rst2::kanMX6 tup11::ura4 tup12::ura4*	[21]
SPH147	*h^+^ ura4-D18 tup11::ura4 tup12::ura4*	[17, 18]
SPH156	*h^+^ ade6-M26 ura4-D18 php5::kanMX6 tup11::ura4 tup12::ura4*	[22]
SPH157	*h^+^ ade6-M26 leu1-32 ura4-D18 php5::kanMX6 rst2-3flag<<kanMX6*	[22]
SPH214	*h^−^ ade6-M26 ura4-D18 his3-D1 atf1::ura4 tup11::ura4 tup12::ura4*	This study
SPH861	*h^+^ ura4-D18 php5::KanMX6 fbp1UAS1,2-del 46bp*	This study
SPH877	*h^+^ ura4-D18 php5::KanMX6 fbp1UAS1,2-del 100bp*	This study
SPH944	*h^+^ ade6-M26 ura4-D18 php5::kanMX6 tup11::ura4 tup12::ura4 rst2-3flag<<hphMX6*	This study
SPH960	*h^+^ ade6-M26 ura4-D18 leu1-32 tup11::ura4 tup12::ura4 php5::KanMX6 fbp1UAS1,2 -del 46bp*	This study
SPH962	*h^+^ ura4-D18 leu1-32 tup11::ura4 tup12::ura4 php5::KanMX6 fbp1UAS1,2-del 100bp*	This study
SPH984	*h^+^ ura4-D18 tup11::ura4 tup12::ura4 php5::KanMX6 fbp1UAS1,2 -del 46bp rst2-3flag<<hphMX6*	This study

### Northern blotting and chromatin immunoprecipitation

Northern blotting was performed as described previously [20]. DNA probes for *fbp1, ght3, mug14*, and *cam1* were amplified by PCR using primer sets 5′*-*CGCCGATACAATCAGAAGC-3′/5′*-*GGATGCTGCAAACTCATCG-3′, 5′*-*TCAATGTCCGGATGGTTGCA-3′/5′-CCCATATTGATACAGGCTGC-3′, 5′*-*GGCTAAAGTAACTGGATTGCC-3′/5′*-*CAATGGCTTTCCCAAAGCTG-3′, and 5′*-*CTACCCGTAACCTTACAG-3′/5′-TGGAAGAAATGACACGAG-3′, respectively. The *cam1* gene was quantified as a loading control as previously conducted [34, 35]. Chromatin immunoprecipitation (ChIP) was performed as described previously [20] except for the DNA shearing method. In this study, we performed DNA shearing using a Picoruptor (Diagenode, Seraing, Belgium) for more extensive shearing compared to the conditions previously used [20], to shear the DNA into ∼200 bp fragments, allowing us to analyze the changes of TF’s distribution in *fbp1* upstream region (Supplementary Fig. S2). Anti-Atf1 (Ab18123, Abcam, Cambridge, UK) and anti-FLAG M2 antibodies (F1804, Sigma–Aldrich, STL, USA) were used for the ChIP analysis. DNA concentrations were quantified using Thermal Cycler Dice Real Time (Takara Bio, Shiga, Japan) and THUNDERBIRD^®^ SYBR qPCR Mix (TOYOBO, Osaka, Japan). The following primer sets, 5′-GGGATGAAAACAATCAACCTC-3′/5′-GGAATGCAGCAACGAAAATC-3′, 5′-GGGTGGAATGAGTCCGC-3′/5′-GTTCCGCGAATCATAAGCC-3′, 5′-GGGATGAAAACAATCAACCTC-3′/5′-GAAATTCGATGATGAGTACTAATG-3′, 5′-GTAGTTTTCATTGCAAGCCC-3′/5′-GTTCCGCGAATCATAAGCC-3′, and 5′-GCACAGTCGTTGTACAAATTCGTATTCCC-3′/5′-ACGATTCTAAACGCCTCTTGTTACGATCC-3′ were used for quantitative PCR analysis for UAS1, UAS2, UAS1 in the UAS1-2 truncation experiment, UAS2 in the UAS1-2 truncation experiment and *prp3*, respectively.

### Construction of tup11∆/tup12∆/php5∆ 46 bp (UAS1–UAS2) and tup11∆/tup12∆/php5∆ 100 bp (UAS1–UAS2) cells with sequence deletions between UAS1 and UAS2

*tup11∆/tup12∆/php5∆* cells were obtained from the ascospore of tetrad dissection crossed between *tup11∆/tup12* and *php5∆* cells [19]. To construct *46 bp (UAS1–UAS2) and 100 bp (UAS1–UAS2)* cells, we deleted regions −1132 to −552 and −1132 to −606 (relative to the first A in the ORF as +1) by polymerase chain reaction (PCR), using the primer sets 5′-GTAGTTTTCATTGCAAGCCC-3′/5′-GAAATTCGATGATGAGTACTAATG-3′ and 5′-CTTCGTTGAATTGCAGTATG-3′/5′-GAAATTCGATGATGAGTACTAATG-3′. We used the *fbp1* upstream region cloned in pCR^®^-Blunt II-TOPO^®^ (Thermo Fisher Scientific, MA, USA) as a template. PCR products were phosphorylated by T4 Polynucleotide Kinase (Takara Bio, Shiga, Japan) and ligated using T4 DNA Ligase (TOYOBO, Osaka, Japan). Fission yeast cells carrying the *ura4* marker gene in the *fbp1* promoter were transformed with these plasmid constructs and selected for uracil auxotrophy on medium containing 5-fluoroorotic acid (5FOA).

### Western blotting

Cultured cells (3 × 10^7^ cells) were resuspended in 300 μl of 20% trichloroacetic acid. After the disruption of the cells using a Multi-Beads Shocker (Yasuikikai, Osaka, Japan) and centrifugation, the supernatant was removed. Precipitated proteins were resuspended in 100 μl of 2 × sodium dodecyl sulphate (SDS) sample buffer [100 mM Tris–Cl (pH 6.8), 200 mM dithiothreitol (DTT), 20% glycerol, 10% 2-mercaptoethanol, 4% SDS, and 0.2% bromophenol blue] and 100 μl of 1 M Tris–Cl (pH 8.8). The protein samples were boiled at 100°C for 5 min, then used for sodium dodecyl sulphate–polyacrylamide gel electrophoresis. To detect Atf1, Rst2, and H3, Anti-Atf1 (Abcam, Cambridge, UK), anti-FLAG M2 antibodies (Sigma–Aldrich, STL, USA), and anti-H3 (Abcam, Cambridge, UK) were used respectively.

### Transcriptome analyses of *S. pombe*

*Schizosaccharomyces pombe* strains (wild-type, *tup11*∆/*tup12*∆, and *tup11∆/tup12∆/php5∆* cells) were cultured in glucose rich, glucose starvation, or nitrogen starvation conditions for 2 h as described in cell culture section. Total RNA samples were prepared as described previously [36]. The library preparations and following next generation sequence (NGS) analyses were conducted by Nippon Genetics (Tokyo Japan). These libraries were nonstrand-specific and were prepared using poly(A) selection. The raw NGS data are available at Gene Expression Omnibus (https://www.ncbi.nlm.nih.gov/geo/, accession number: GSE342430). Raw data of NGS were mapped to the *S. pombe* genome (ASM294v2.30) using hisat2. Reads mapped to each gene were counted using featureCounts (https://subread.sourceforge.net/featureCounts.html). Gene annotation data that were available on 4 January 2025, were downloaded from PomBase. Only protein-coding genes were considered. The read counts were normalized using transcripts per million (TPM). We considered genes that were upregulated after glucose starvation by more than two-fold compared with glucose rich conditions as glucose-responding genes, excluding high-expressed genes in which the TPM under glucose-rich conditions was larger than 100 and low-expressed genes in which the TPM under glucose starvation conditions was <100. We further selected genes exhibiting over two-fold induction in nitrogen starvation conditions in *tup11*∆/*tup12*∆ compared to wild-type cells, which are dependent on Php5 (over two-fold reductions in *tup11∆/tup12∆/php5∆* compared to *tup11*∆/*tup12*∆ cells).

### Visualization of ChIP-seq data

Raw data of sequential ChIP-seq of Atf1-Rst2 (accession number: DRA011814 as previously described) [23] were mapped to the *S. pombe* genome (ASM294v2.30) using bowtie2 (https://bowtie-bio.sourceforge.net/bowtie2/index.shtml). For the visualization, all mapped data were normalized using counts per million, binned using a 25 bp window, and IP scores were divided by that of Input. The normalized data were visualized as heatmaps using R (https://www.r-project.org/). We provided UCSC genome browser session showing localization of Atf1-Rst2 around *fbp1, mug14, dak2, ght3*, and *gld1* genes as follows, respectively. *fbp1*: https://genome.ucsc.edu/s/seibutsukagaku2025/share_eH1v9yNC, *mug14*: https://genome.ucsc.edu/s/seibutsukagaku2025/share_5RVdf2z2, *dak2*: https://genome.ucsc.edu/s/seibutsukagaku2025/share_AAFZZDsw, *ght3*: https://genome.ucsc.edu/s/seibutsukagaku2025/share_6A8vdq3O, and *gld1*: https://genome.ucsc.edu/s/seibutsukagaku2025/share_1fKbA8WB.

### Statistical analysis

Statistical analyses were performed using R as described in the figure legend. The two-way analysis of variance (ANOVA) was performed with genotype and stress condition as factors using the aov function. In the ANOVA of the ChIP experiment using *tup11∆/tup12∆* cells (Figs 2B and 3B), each fold enrichment value was log_2_-transformed before statistical analysis to stabilize variance and better satisfy the assumptions of ANOVA.

## Results

### Nonspecific fbp1 transcription in tup11∆/tup12∆ cells under nitrogen starvation bypasses the mlonRNA requirement

To elucidate the mechanisms underlying stress-specific transcriptional responses, we examined the aberrant induction of *fbp1* in *tup11∆/tup12∆* cells. As previously reported [37], *tup11∆/tup12∆* cells, but not wild-type cells, display pronounced transcriptional activation of *fbp1* in response to nitrogen starvation, a stress unrelated to glucose depletion. We therefore compared the transcriptional dynamics of *fbp1* between glucose starvation (the specific inducer) and nitrogen starvation (aberrant *fbp1* expression inducer in *tup11∆/tup12∆* cells).

**Figure 1.**
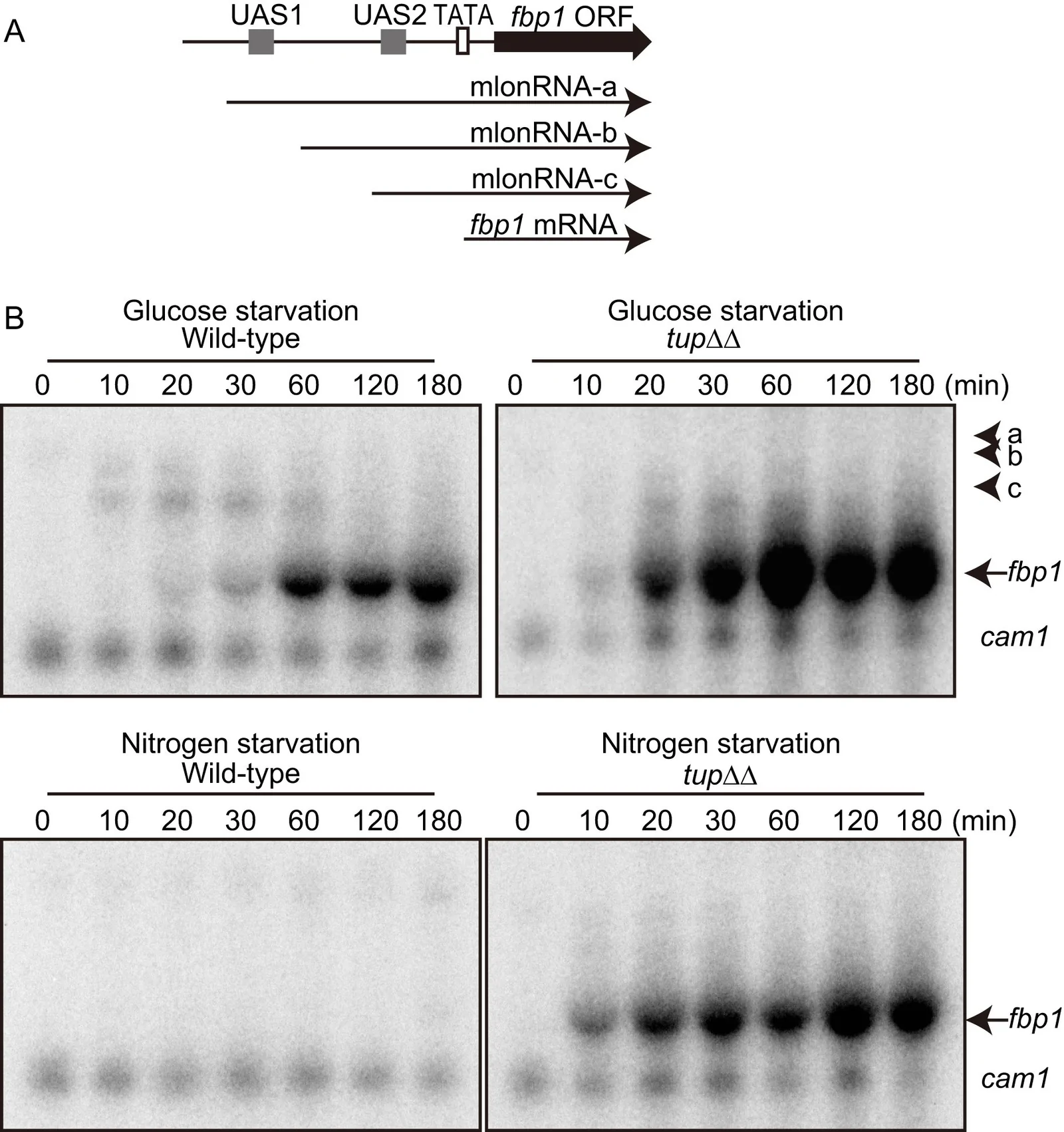
Aberrant *fbp1* induction in *tup11∆/tup12∆* cells upon nitrogen starvation stress bypasses the requirement for mlonRNA expression. (**A**) Schematic illustration of *fbp1* gene upstream region. The structures of the transcripts of mlonRNAs (a, b, and c) and mRNA of *fbp1* are shown. (**B**) Representative image showing the transcription pattern of the *fbp1* gene in wild-type cells (left) and *tup11∆/tup12∆* cells (right) at indicated times after glucose starvation (upper) and nitrogen starvation (lower). Arrow heads labeled a, b, and c identify the mlonRNA-a, mlonRNA-b, and mlonRNA-c, respectively. Arrow represents *fbp1* mRNA. *cam1* was probed as a loading control. The experiment was repeated twice (*n* = 2).

As previously demonstrated [21], in wild-type cells subjected to glucose starvation, *fbp1* activation occurs through stepwise expression of metabolic stress-induced long noncoding RNAs (mlonRNAs) whose transcriptional initiation sites progressively shift from upstream to downstream regions (Fig. 1A and B). Expression of mlonRNA-a precedes those of mlonRNA-b and -c, culminating in robust *fbp1* mRNA activation after 60 min of glucose starvation (Fig. 1A and B). The actual shifts of transcriptional initiation sites have been demonstrated previously [21]. By contrast, neither mlonRNAs nor *fbp1* mRNA is expressed upon nitrogen starvation in wild-type fission yeast cells (Fig. 1B), indicating that wild-type cells can discriminate between nitrogen- and glucose-starvation stress. Strikingly, *tup11∆/tup12∆* cells exhibit significant expression of *fbp1* mRNA within 10 min of nitrogen starvation without detectable mlonRNA expression (Fig. 1B). Thus, in the absence of Tup11/12, *fbp1* activation upon nitrogen starvation bypasses the requirement for mlonRNA transcription-dependent chromatin remodeling, which is normally prerequisite for transcriptional activation via TF binding [21].

### Atf1 and Rst2 are required for aberrant fbp1 activation upon nitrogen starvation in tup11∆/tup12∆ cells

**Figure 2.**
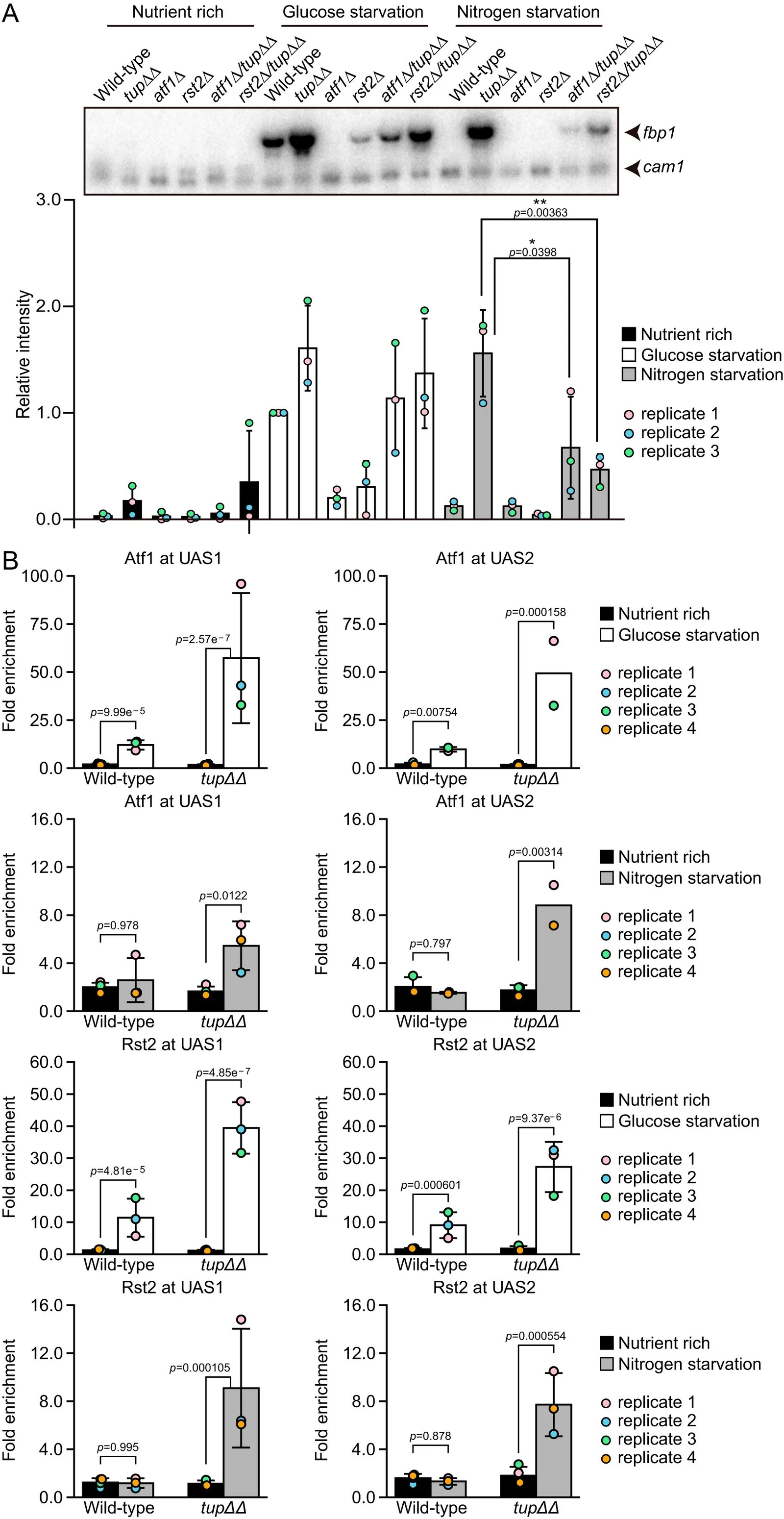
Atf1 and Rst2 are required for aberrant *fbp1* induction upon nitrogen starvation in *tup11∆/tup12∆* cells. (**A**) Representative image showing the transcription of the *fbp1* gene in indicated cells in normal (glucose-rich), glucose starvation, and nitrogen starvation conditions. Indicated fission yeast cells were cultured in YER (nutrient rich), and then transferred to YED (glucose starvation), or MM − N (nitrogen starvation) medium and further cultured for 2 h. Relative levels of *fbp1* induction were quantified from three independent experiments (*n* = 3). Error bars represent the standard deviations (SD). (**B**) The bindings of Atf1 and Rst2 in glucose starvation or nitrogen starvation conditions in wild-type and *tup11∆/tup12∆* cells were examined by ChIP analyses as described in the ‘Materials and methods’ section. Fold enrichment normalized by the value at the *prp3* locus is presented. Error bars represent the SD from independent experiments (*n* = 2 for Atf1 at UAS2 in YED and MM − N, *n* = 4 for Atf1 and Rst2 at UAS1 and UAS2 in YER, *n* = 3 for another conditions, see S7_raw_data for Fig. 2B). *P*-values were calculated by two-way ANOVA followed by Tukey’s honestly significant difference (HSD) test. Each fold enrichment value was log_2_-transformed before statistical analysis (see the ‘Materials and methods’ section).

Since aberrant *fbp1* activation upon nitrogen starvation in *tup11∆/tup12∆* cells bypasses the requirement of prerequisite mlonRNA expression, we next asked if this aberrant *fbp1* activation in *tup11∆/tup12∆* cells also bypasses the requirement of two essential TFs, Atf1 and Rst2. To this end, we examined the impact of the loss of either Atf1 or Rst2 on the *fbp1* activation in *tup11∆/tup12∆* cells upon nitrogen starvation. Deletion of either *atf1* or *rst2* gene markedly reduces *fbp1* induction upon nitrogen starvation stress in *tup11∆/tup12∆* cells (Fig. 2A), indicating that both factors remain indispensable even in the absence of Tup11/12. ChIP analyses revealed that, in wild-type cells, Atf1 and Rst2 bind to both UAS1 and UAS2 only under glucose-starved conditions, as previously observed [22] (Fig. 2B). In contrast, both factors bind to these regions in *tup11∆/tup12∆* cells, but not in wild-type cells, upon nitrogen starvation (Fig. 2B). These results indicate that Atf1 and Rst2 can bind to their essential *cis-*elements for *fbp1* induction upon nitrogen starvation conditions in the absence of Tup11/12. Thus, Tup11/12 act to destabilize Atf1 and Rst2 binding under a noninduction condition, thereby preventing inappropriate *fbp1* activation.

### Php5 is essential for aberrant fbp1 induction upon nitrogen starvation in tup11∆/tup12∆ cells

**Figure 3.**
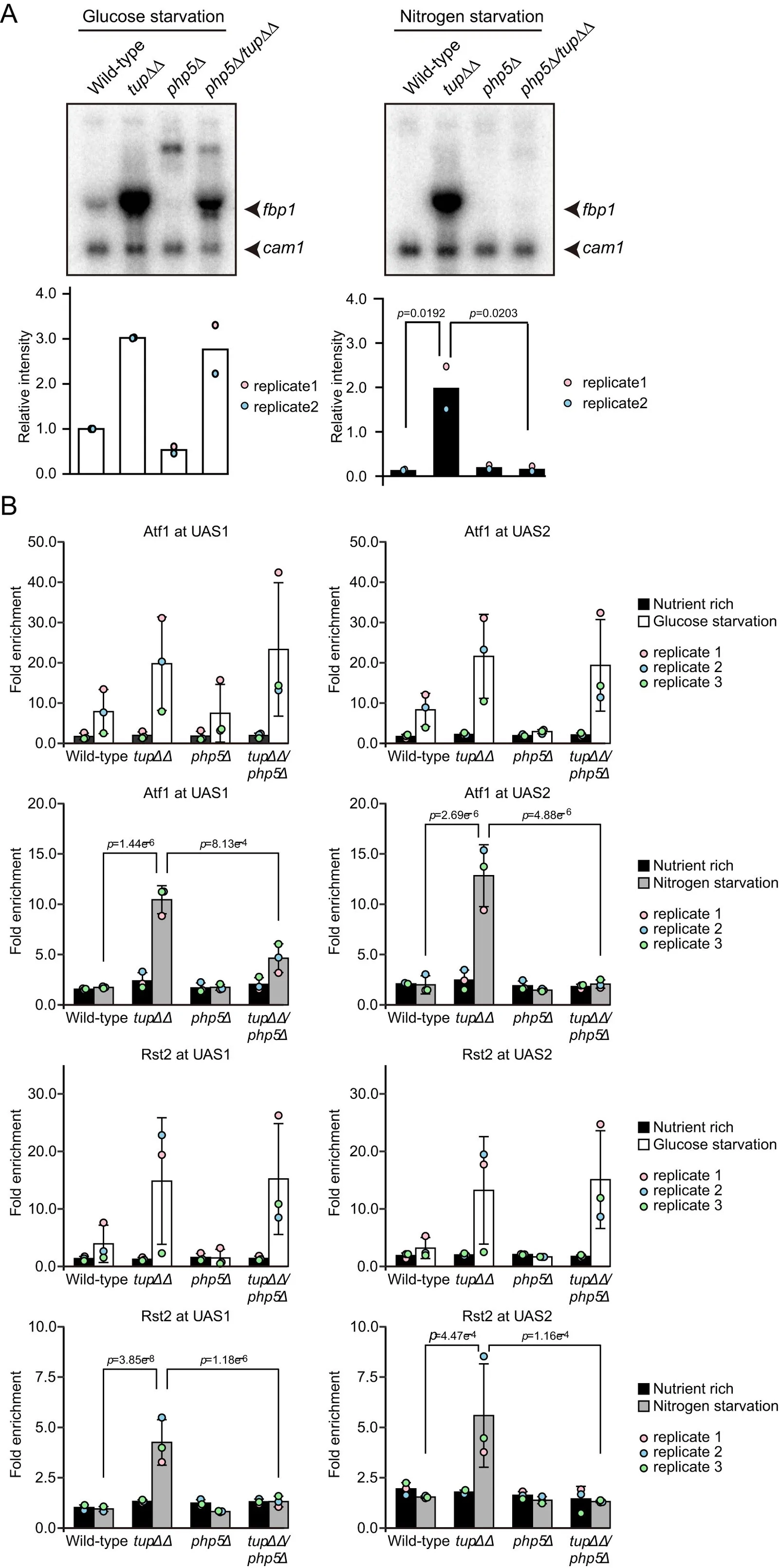
Php5 counteracts Tup11/12-mediated suppression of Atf1 and Rst2 binding to *fbp1* upstream *cis-*elements. (**A**) Representative image showing the transcription of the *fbp1* gene in indicated cells upon glucose starvation and nitrogen starvation. Indicated cells were cultured in YED or MM − N medium for 2 h. Relative levels of *fbp1* induction were quantified from two independent experiments (*n* = 2). *P*-values were calculated by two-way ANOVA followed by Tukey’s HSD test. (**B**) The binding of Atf1 and Rst2 upon glucose starvation or nitrogen starvation in indicated cells was examined by ChIP analyses as described in the ‘Materials and methods’ section. Fold enrichments normalized by the value at the *prp3* locus are presented. Error bars represent SD from three independent experiments (*n* = 3). *P*-values were calculated by two-way ANOVA followed by Tukey’s HSD test. Each fold enrichment value was log_2_-transformed before statistical analysis (see the ‘Materials and methods’ section).

A highly conserved CBF/NF-Y type TF, Php5, promotes *fbp1* activation by mediating DNA-loop formation, which juxtaposes UAS1 and UAS2, facilitating cooperative binding of Atf1 and Rst2 [22]. To assess whether Php5 also contributes to the aberrant *fbp1* induction upon nitrogen starvation, we examined *php5∆* cells. In *php5∆* cells, upon glucose starvation, *fbp1* mRNA expression is impaired, but a longer transcript accumulates (Fig. 3A). This longer transcript is mlonRNA-c, which is produced due to the arrest of the cascade expression (mlonRNA-a, mlonRNA-b, mlonRNA-c, and mRNA) following the expression of mlonRNA-c [22]. The loss of Php5 in *tup11∆/tup12∆* cells also abrogates *fbp1* induction upon nitrogen starvation (Fig. 3A). These data indicate that Php5 is required for both specific and aberrant *fbp1* induction. ChIP analysis revealed that the loss of Php5 markedly reduces Atf1 and Rst2 binding in wild-type cells upon glucose starvation (Fig. 3B, white bars). Interestingly, in *tup11∆/tup12∆/php5∆* cells, significant binding of Atf1 and Rst2 is detected around the alternative site (UAS2 and UAS1 for Atf1 and Rst2, respectively) upon glucose starvation, suggesting that these TFs can bind to binding sites other than their *cis-*element even in the absence of a Php5 mediated-DNA loop between UAS1 and UAS2. This might be attributable to the presence of a CT-rich STRE like sequence (Supplementary Fig. S3A) [23] and CRE-like sequence (Supplementary Fig. S3B) upstream of the UAS1 and UAS2, respectively. Atf1 might be able to bind directly to this CRE-like sequence near UAS2 sequence without transition from UAS1 via a loop structure, in the absence of Tup11/12, similarly to how Rst2 binds to UAS1 as previously demonstrated [22]. More importantly, the loss of Php5 in nitrogen starved *tup11∆/tup12∆* cells (nonspecific conditions) critically reduces the binding of Atf1 and Rst2 (Fig. 3B, gray bars). These results highlight the critical role of Php5 in stabilizing TF binding in aberrant *fbp1* induction upon nitrogen starvation in *tup11∆/tup12∆* cells. Moreover, these findings suggest that Php5-mediated TF stabilization, likely through close proximity between UAS1 and UAS2, counteracts Tup11/12-mediated TF destabilization in the *fbp1* upstream region.

### Spatial proximity of UAS1 and UAS2 is crucial for TF stability

**Figure 4.**
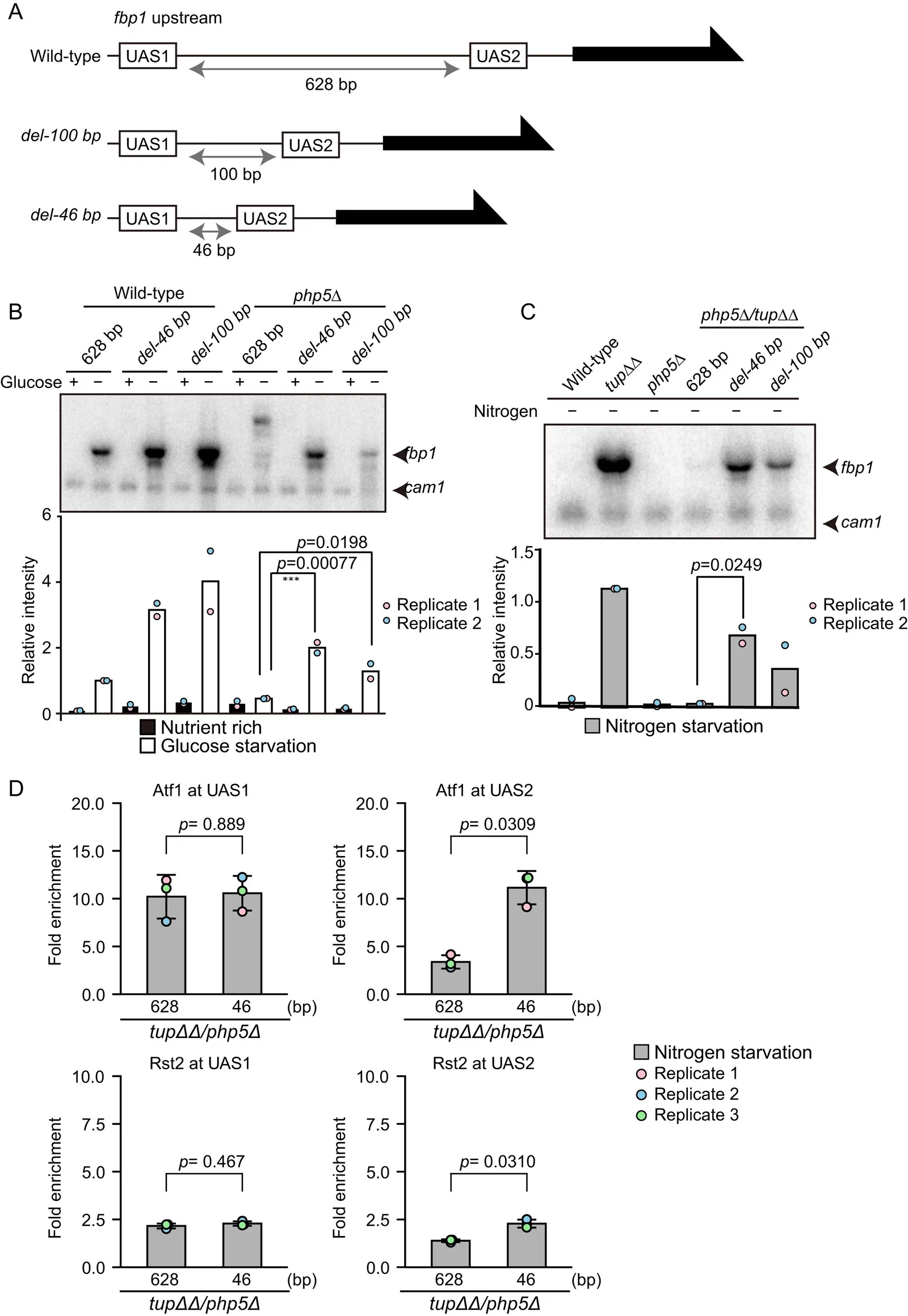
Important role of the close proximity between UAS1 and UAS2 in *fbp1* induction. (**A**) Schematic drawing of the region upstream from *fbp1* containing UAS1 and UAS2. The distance between UAS1 and UAS2 is artificially changed to 46 bp or 100 bp, while the spacing between these sites is 628 bp in wild-type cells. (**B, C**) Representative images showing the transcription of the *fbp1* gene in indicated cells in glucose starvation (**B**) and nitrogen starvation (**C**) conditions. Indicated cells were cultured in YED or MM − N medium for 2 h. Quantitative data of *fbp1* induction upon glucose starvation (**B**) and nitrogen starvation (**C**) are presented under the representative blot images. The experiment was repeated twice (*n* = 2). *P*-values were calculated by one-way ANOVA followed by Tukey’s HSD test. (**D**) The binding of Atf1 upon nitrogen starvation in indicated cells was examined by ChIP analyses as described in the ‘Materials and methods’ section. Fold enrichments normalized by the value at the *prp3* locus are presented. Error bars represent SD from three independent experiments (*n* = 3). *P*-values were calculated by *t*-test.

To test the hypothesis that proximity between UAS1 and UAS2 contributes to reciprocal TFs’ stabilization and is ultimately required for the aberrant *fbp1* activation upon nitrogen starvation in *tup11∆/tup12∆* cells, we manipulated the distance between UAS1 and UAS2 by deleting the intervening sequences between these two elements (Fig. 4A). We placed UAS1 and UAS2 within 46, 100, and 628 bp (without deletion). Since the Atf1 and Rst2 binding sequences in the UAS1 region are naturally separated by 46 bp (Supplementary Fig. S3A), and in this configuration, Atf1 and Rst2 reciprocally stabilize each other [23], we placed these *cis-*elements in a similar proximity (Fig. 4A). Placing UAS1 and UAS2 within 46 bp bypassed requirement of Php5 in specific *fbp1* induction (glucose-starved *tup11^+^/tup12^+^* cells), indicating that Php5 indeed stabilizes TFs via a loop structure-mediated closed proximity (Fig. 4B). More importantly, placing UAS1 and UAS2 within 46 bp proximity significantly restores the aberrant *fbp1* induction in *tup11∆/tup12∆/php5∆* cells (Fig. 4C). Consistently, in the conditions of aberrant *fbp1* induction (upon nitrogen starvation in *tup11∆/tup12∆* cells), the loss of Php5 critically impairs the binding of Atf1 and Rst2 to UAS2 (Fig. 3B), while placing UAS1 and UAS2 within 46 bp proximity significantly restores these bindings (Fig. 4D). These data indicate that TFs reciprocally stabilize each other in close proximity. Taken together, these results demonstrate that the response to glucose starvation is regulated through the counteracting mechanisms of TF stabilization via the close proximity between UAS1 and UAS2 by Php5 and TF destabilization by Tup11/12.

### Distinct activation status of Atf1 and Rst2 in response to glucose starvation and nitrogen starvation

**Figure 5.**
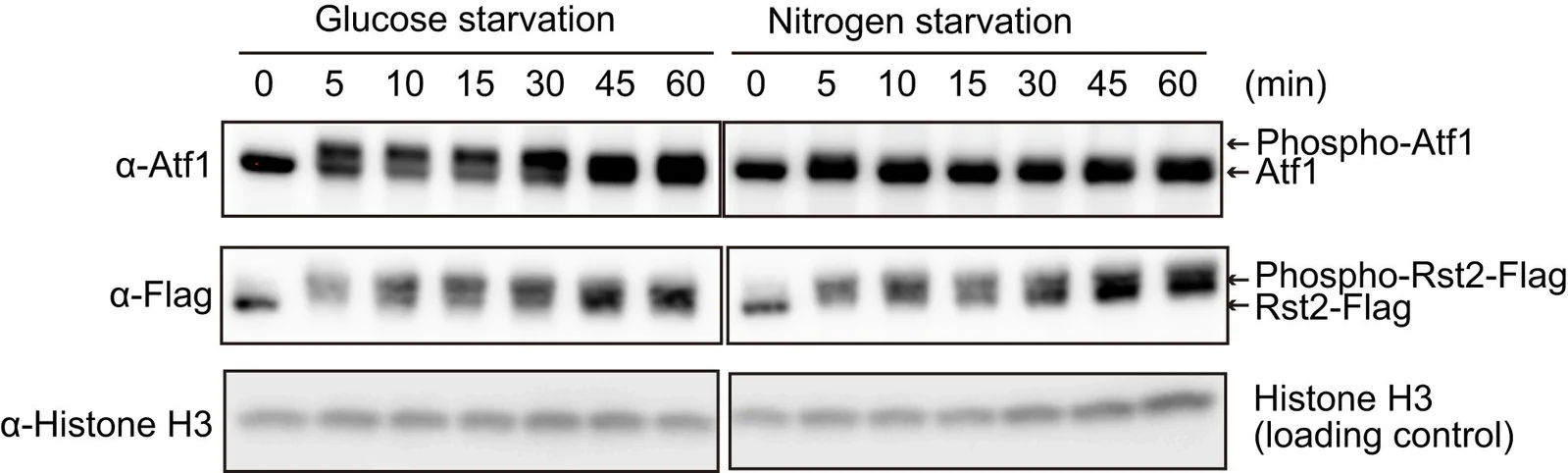
Insufficient and late activation status of Atf1 upon nitrogen starvation. Representative images showing the phosphorylation status of Atf1 and Rst2. Wild-type fission yeast cells were cultured in YED or MM − N for the indicated time. Arrows indicate the bands corresponding to Atf1, phosphorylated Atf1, Rst2, and phosphorylated Rst2. Histone H3 is shown as a loading control. These experiments were repeated twice and a similar trend was confirmed (*n* = 2).

To understand how the above dual regulations—Php5-mediated stabilization and Tup11/12-mediated destabilization of TFs—decode the different kinds of external stresses, we monitored the phosphorylation status of Atf1 and Rst2 in response to glucose starvation or nitrogen starvation. The phosphorylation status of Atf1 and Rst2 can be detectable as a band shift [10, 37]. Upon glucose starvation, both Atf1 and Rst2 are rapidly phosphorylated within 5 min, maintaining this highly active state up to 60 min (Fig. 5). In marked contrast, nitrogen starvation induces comparable Rst2 phosphorylation, whereas phosphorylation of Atf1 is markedly weaker and delayed (Fig. 5). These observations suggest that the activation status of TFs upon nitrogen starvation is weaker and slower than that upon glucose starvation. Taken together, these results suggest that the counteracting mechanism by Php5-mediated TF stabilization and Tup11/12-mediated TF destabilization interprets differential activation states of Atf1 and Rst2 to confer stress-specific transcriptional response.

### The dual regulatory mechanism—TF stabilization by Php5-mediated and TF destabilization by Tup11/12—regulates other genes within the fission yeast genome

**Figure 6.**
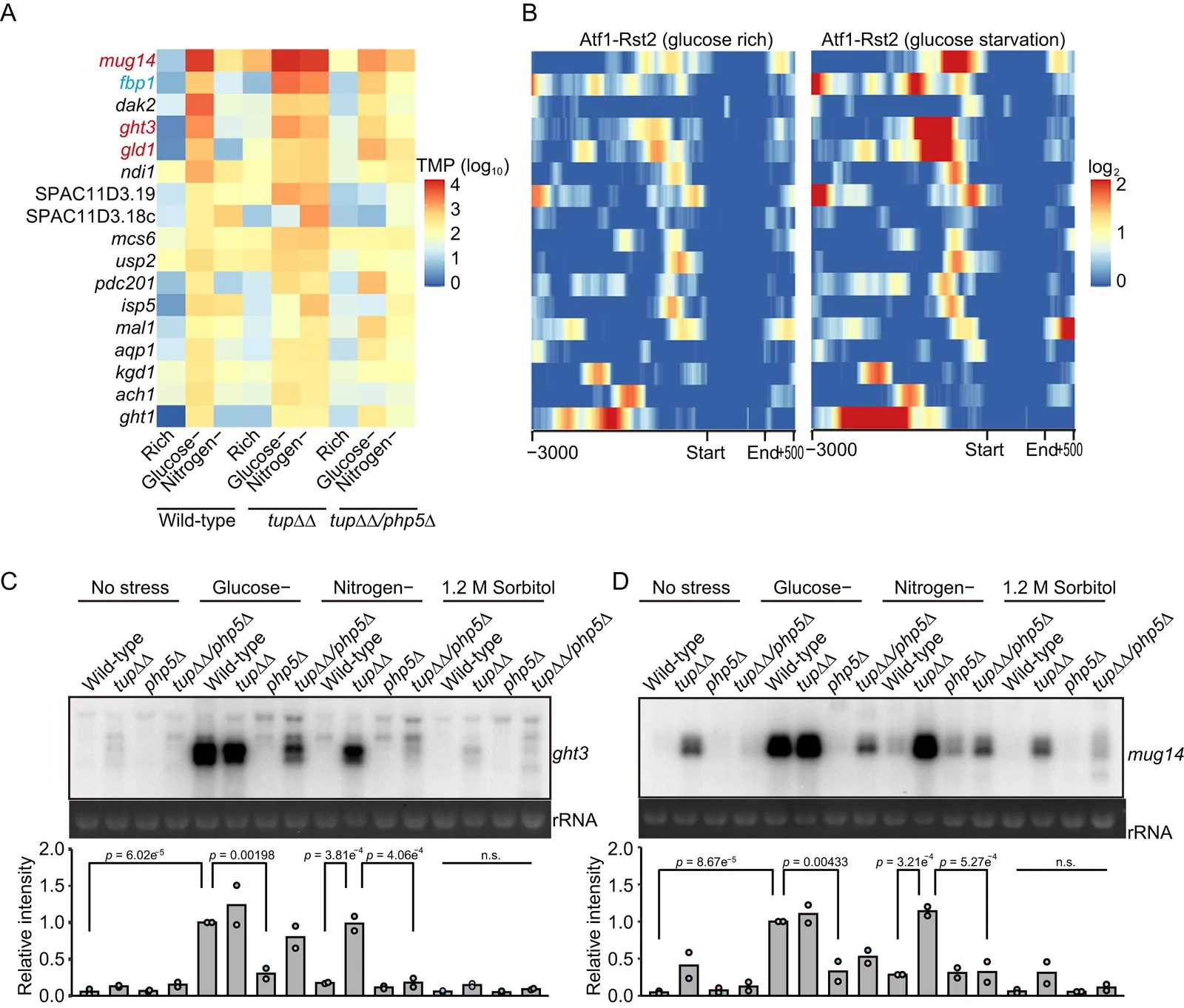
Conserved counteractive regulation by Tup11/12 and Php5. (**A**) 17 genes whose expression patterns are similar to that of *fbp1* are shown (left). Genes whose expression are induced during glucose starvation (over two-fold induction in glucose starvation compared to nutrient rich conditions), and whose unspecific induction upon nitrogen starvation are observed in *tup11∆/tup12∆* (over two-fold induction in *tup11∆/tup12∆* compared to wild-type cells upon nitrogen starvation), and such unspecific induction are dependent on Php5 (over two-fold reduction in *tup11∆/tup12∆/php5∆* compared to *tup11∆/tup12∆* upon nitrogen starvation). Colors represent the TPM showing number of read counts in each gene. Gene names highlighted by red and blue indicate genes exhibiting promoter binding of Atf1 and Rst2 in glucose starvation conditions and the *fbp1* gene, respectively. (**B**) The status of co-binding of Atf1 and Rst2 in the promoter region of genes presented in A (left). Colors represent log_2_-transformed IP/INPUT of Atf1-Rst2 sequential ChIP-seq. (**C, D**) Representative images showing the expression pattern of *ght3* (**C**) and *mug14* (**D**) under nutrient rich (YER), glucose starvation (YED), nitrogen starvation (MM − N), or osmotic stress (YE + 1.2M sorbitol) conditions for 2 h are presented. Relative levels of *ght3* and *mug14* induction were quantified from two independent experiments (*n* = 2). *P*-values were calculated by two-way ANOVA followed by Tukey’s HSD test.

We next sought to determine whether the regulatory system observed in the *fbp1* gene, which governs stress specificity, controls other stress selective gene responses in fission yeast genome. To this end, we searched for the genes exhibiting an expression pattern similar to the *fbp1* gene by transcriptome analyses. We compared the transcriptome of wild-type, *tup11∆/tup12∆*, and *tup11∆/tup12∆/php5∆* cells under nutrient rich, glucose starvation, and nitrogen starvation conditions. We looked for genes whose expression are induced during glucose starvation (over two-fold induction in glucose starvation compared to glucose rich conditions), and whose aberrant induction upon nitrogen starvation is observed in *tup11∆/tup12∆* (over two-fold induction in *tup11∆/tup12∆* compared to wild-type cells upon nitrogen starvation), and whose aberrant induction is dependent on Php5 (over two-fold reduction in *tup11∆/tup12∆/php5∆* compared to *tup11∆/tup12∆* upon nitrogen starvation). Based on these criteria, we identified seventeen genes including the *fbp1* gene (Fig. 6A, blue). Integration of previously published sequential ChIP-seq of Atf1 and Rst2 [23] revealed that three of these genes—*mug14, ght3*, and *gld1*—show substantial Atf1 and Rst2 binding within 1 kb upstream of their ORF regions in response to glucose starvation (Fig. 6B). We then examined the expression pattern of *mug14* and *ght3* genes under nutrient rich, glucose starvation, and nitrogen starvation conditions by northern blot analysis. Both genes are massively activated by glucose starvation in wild-type cells in a Php5-dependent manner (Fig. 6C and D). Moreover, both genes exhibit aberrant induction under nitrogen starvation conditions in *tup11∆/tup12∆* cells, which is abolished in *tup11∆/tup12∆/php5∆* cells (Fig. 6C and D). A similar trend was also observed in the case of osmotic stress. However, the degree of the aberrant induction levels is not pronounced and is not statistically significant (Fig. 6C and D). These data indicate that the glucose starvation stress-specific responses of *mug14* and *ght3* genes are governed by mechanisms similar to that of the *fbp1* gene. Analysis of the promoter sequences of these genes revealed that *ght3* harbors two composite motifs analogous to *fbp1*, containing closely spaced CRE-core (TGACGT)-like and STRE (CCCC[G/T]C)-like sequences within about 50 bp, while *mug14* contains four STRE-like sequences but no canonical CRE-core sequence (Supplementary Fig. S4). Given that ATF family proteins can bind to incomplete CREs depending on the surrounding sequence [38], it is possible that Atf1 binds to regions lacking CRE-core. These findings indicate that the dual mechanism of Tup11/12-mediated TF destabilization and Php5-mediated stabilization governs a multiple stress-responsive genes.

### Tup11/12 and Php5 cooperatively regulate cellular adaptation to environmental stresses

**Figure 7.**
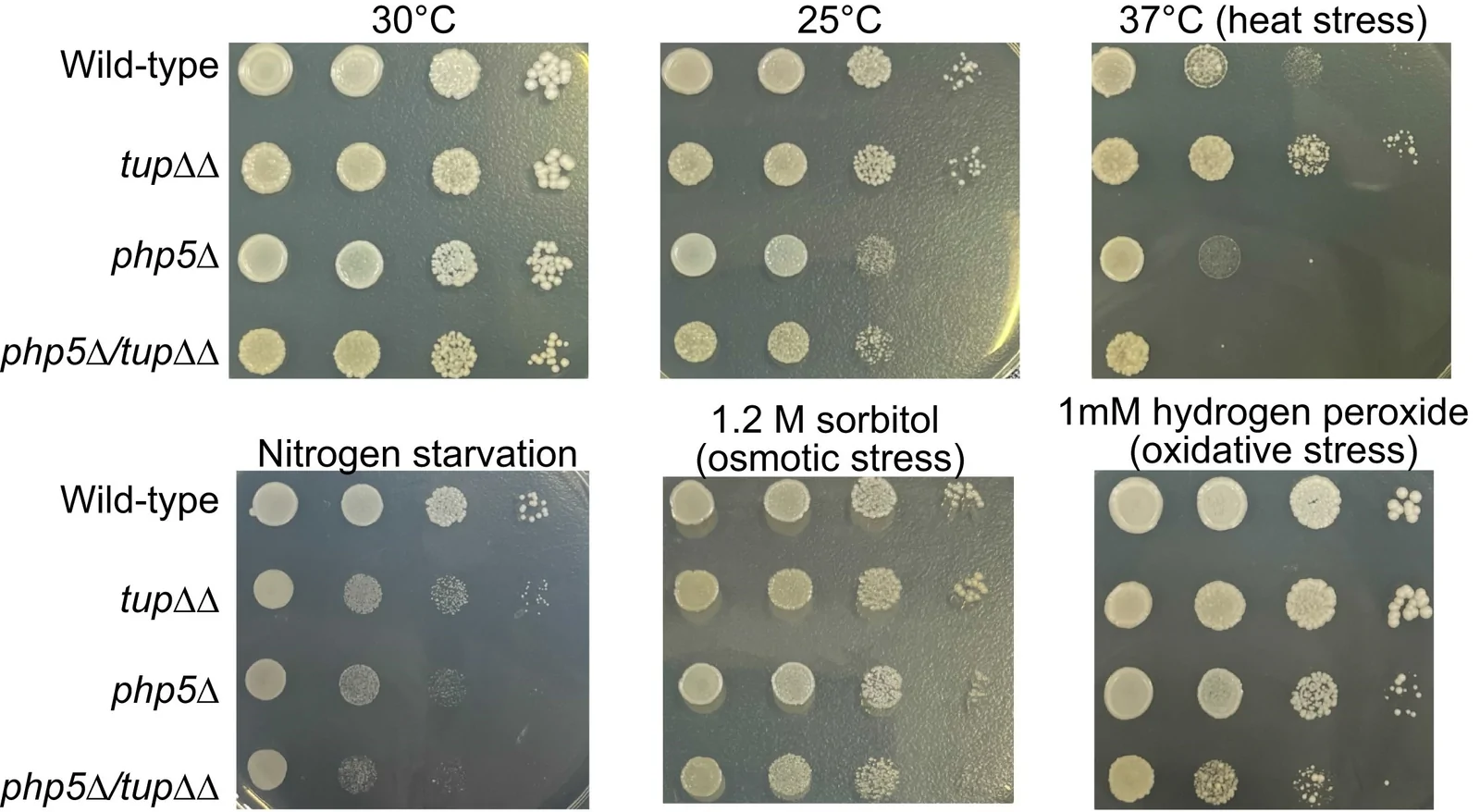
Pivotal role of Tup11/12 and Php5 in response to stress conditions by fission yeast. Wild-type, *tup11∆/tup12∆, php5∆*, and *tup11∆/tup12∆/php5∆* cells were cultured in YE liquid medium and harvested at log phase. These cells were suspended in distilled water, then serially diluted by 10-fold and spotted on YE medium and exposed indicated stress conditions. The experiment was repeated two times, and the same results were obtained.

Finally, to assess the physiological relevance of this regulatory system in cellular adaptation to the external stresses, we evaluated the sensitivities of wild-type, *tup11∆/tup12∆, php5∆*, and *tup11∆/tup12∆/php5∆* cells under various environmental challenges. Under 25°C culture conditions, which is slightly lower temperature than optimal temperature for fission yeast (30°C), *php5∆* cells exhibit mildly reduced survival, without synergistic effects with *tup11∆/tup12∆* (Fig. 7). In contrast, under heat and nitrogen starvation stresses, the loss of Tup11/12 exacerbates the sensitivity of *php5∆* cells, revealing a synergistic requirement for both factors (Fig. 7). Under osmotic and oxidative stresses, both *tup11∆/tup12∆* and *php5∆* cells remain relatively tolerant, however simultaneous loss of both Tup11/12 and Php5 causes marked sensitivity (Fig. 7). Collectively, these results highlight a pivotal role of Tup11/12 and Php5 in maintaining transcriptional fidelity and ensuring robust cellular adaptation to diverse environmental stresses.

## Discussion

In this study, we investigated the mechanisms underlying stress-specific transcriptional response to external stresses. We found that *fbp1* induction upon nitrogen starvation stress (nonspecific condition) is aberrantly triggered in *tup11∆/tup12∆* cells by bypassing the prerequisite mlonRNA transcription, which normally induces chromatin remodeling to permit binding of TFs to their binding sites [21] (Fig. 1). This aberrant induction is mediated by two TFs, Atf1 and Rst2, both essential for physiological *fbp1* activation upon glucose starvation [17] (Fig. 2). In the absence of Tup11/12, these two TFs aberrantly bind to upstream *cis*-elements of the *fbp1* gene upon nitrogen starvation (Fig. 2), suggesting that Tup11/12 normally act to destabilize inappropriate TF binding. Furthermore, the aberrant *fbp1* activation upon nitrogen starvation observed in *tup11∆/tup12∆* cells is diminished by the loss of Php5, a CBF/NF-type TF that facilitates the formation of a local DNA loop between two TF binding sites [22], thereby stabilizing TF binding (Fig. 3). These data indicate that Tup11/12 and Php5 exert opposing effects: Tup11/12 promotes TF destabilization, whereas Php5 stabilizes TFs in close proximity. The requirement of Php5 for aberrant *fbp1* activation is bypassed when UAS1 and UAS2 are artificially juxtaposed, confirming that loop-mediated proximity contributes directly to TF stabilization (Fig. 4). Given that activation states of Atf1 and Rst2 under glucose starvation and nitrogen starvation conditions are distinctly different (Fig. 5), such dual-counteractive regulation—Tup11/12-dependent destabilization and Php5-mediated stabilization of TF binding—likely enables discrimination of the external stresses. Transcriptome analyses further revealed that this mechanism extends to other glucose starvation-responsive genes, including *ght3* and *mug14*. (Fig. 6). The *ght3* promoter shares a structural resemblance to that of *fbp1* (Supplementary Fig. S4), with juxtaposed CRE- and STRE-like motifs. Finally, we demonstrated the vital roles of the Tup11/12 and Php5 in enabling fission yeast cells to adapt to various external stresses (Fig. 7), highlighting the importance of the TF binding modulation systems identified in this study in cellular survival upon stress.

In the aberrant *fbp1* activation (in *tup11∆/tup12∆* cells upon nitrogen starvation stress), *fbp1* mRNA is transcribed within 10 min, bypassing the normal requirement for mlonRNA expression (10–30 min of glucose starvation) that precedes mRNA activation (60–180 min of glucose starvation) (Fig. 1). However, this aberrant transcription is mediated through two TFs, Atf1 and Rst2 (Fig. 2), that are also required for glucose starvation-mediated normal *fbp1* transcription. Considering the role of mlonRNA transcription in inducing chromatin remodeling, these results indicate that Tup11/12 protect chromatin to suppress inappropriate TF binding to their binding sites. Consistent with this hypothesis, the chromatin configuration becomes open state upon nonspecific stresses such as osmotic stress or nitrogen starvation [37].

We found that the CBF/NF-Y type TF, Php5, is pivotal for the aberrant *fbp1* response upon nitrogen starvation in *tup11∆/tup12∆*. In response to glucose starvation, Php5, presumably together with CBF subunits Php2 and Php3, induces formation of the local loop between UAS1 and UAS2 to place these sites into close proximity after chromatin remodeling, which in turn promotes translocation of Rst2 from UAS1 to UAS2 and stabilizes its binding by counteracting Tup11/12-mediated suppression of Rst2 binding to UAS2 [22]. Our finding on the role of Php5 in aberrant *fbp1* induction upon nitrogen starvation demonstrates that Php5-mediated close proximity is required for stabilization of inappropriate binding of TFs in the absence of Tup11/12 (Figs 3 and 4). Moreover, this mechanism is also required for normal *fbp1* induction in the presence of Tup11/12 (Fig. 4).

Taken together, we here show a model how signal specificity emerges from the balance between destabilizing and stabilizing forces acting on TF binding. Increasing evidence suggests that TF’s binding selectivity to specific motif sequences cannot explain stress-selective response. One critical regulatory mechanism is mediated by Tup family corepressors (including Tup1 in budding yeast), since Tup proteins stabilize promoter nucleosome positioning and occupancy, thereby contributing to the fine-tuning of transcriptionally plastic genes [29]. Such a protective role by Tup proteins on the positioned nucleosome (condensed chromatin) has been well-documented [18, 39–42] (Supplementary Fig. S5A). The other regulatory mechanism in transcription is mediated though multivalent TF interactions. For example, multivalent TF interactions within super-enhancers cause augmentation of local TF concentration, enhancing transcriptional specificity and robustness [26–28]. Moreover, TF binding in close proximity cause reciprocal stabilization of TFs, leading to cooperative regulation of gene activation [23, 24]. A local loop structure formed by Php5 may increase the effective concentration of the TFs, analogous to condensate-like transcriptional hubs (Supplementary Fig. S5B). In glucose starvation conditions, appropriately activated TFs induce chromatin remodeling through mlonRNA transcription to counteract Tup11/12-mediated suppression [21]. Php5-mediated local loop formation and resultant close proximity between UAS1 and UAS2 might support reciprocal stabilization [22, 23] (Supplementary Fig. S5C). In nitrogen starvation (aberrant *fbp1* expression inducer in *tup11∆/tup12∆* cells), insufficient TF activations (Fig. 5) might not cause mlonRNA induction. This, in turn, fails to reposition nucleosomes (closed chromatin) around regulatory region of *fbp1*, which is maintained by Tup11/12 [37]. As a result of such Tup protein mediated regulation, weak TF binding in the nitrogen starvation conditions might be destabilized. In the absence of the Tup proteins, repressive chromatin cannot be maintained and the weakly bound TFs might be stabilized by reciprocal TF stabilization through proximity which is mediated through local loop between UAS1 and UAS2 formed by Php5 [22] (Supplementary Fig. S5D). In this context, our findings suggest that Tup11/12-mediated destabilization of nonproductive TF binding is counterbalanced by loop-mediated cooperative stabilization of TF assemblies, enabling stress-specific transcriptional responses. Taken together, these data demonstrate that stress specific gene regulation is governed by the balance between destabilizing and stabilizing forces acting on TF binding—TF binding destabilization by Tup11/12 and TF binding stabilization by Php5-mediated loop structure formation (Supplementary Fig. S5E). The loss of both regulatory axes (*tup∆∆/php5∆*) results a in synergistic reduction of cellular viability upon environmental fluctuations (Fig. 7).

Our transcriptome analyses revealed that such counteractive regulation governs expression of other stress-responsive genes such as *ght3* and *mug14* (Fig. 6). Not surprisingly, the *ght3* promoter structure is similar to that of *fbp1*, with both gene promoters possessing CRE-core (TGACGT) and STRE-like (CCCC[G/T]C) sequences nearby. Thus, these counteracting mechanisms—TF binding destabilization by Tup11/12 and TF binding stabilization by Php5—might be a conserved system in fission yeast. Through the sequence analysis of the fission yeast genome, we found 398 CRE-core/STRE-like, 471 CRE-like/STRE-core, and 16 CRE-core/STRE-core sites (total 885 sites), in which CRE (TGACGT) and STRE (CCCC[G/T]C) sequences are located within 30–55 bp of each other. Our previous findings indicated that the proximity of two TF-binding sites contributes to the integration of two independent signal pathways through the reciprocal stabilization of TFs [23]. The presence of >800 closely located TF-binding sites suggests the importance of such regulation. Additional regulation via close proximity mediated by Php5-dependent loop formation might further contribute to distinguishing complex environmental fluctuations.

We have demonstrated a pivotal role of loop-mediated proximity of TF binding sites where Atf1 and Rst2 reciprocally stabilize each other. Similar synergistic action of CREB/ATF and a zinc-finger protein was previously detected in mouse *c-fos* promoter [43]. This gene is repressed by YY1, a zinc-finger protein, mediated by a physical interaction between YY1 and CREB/ATF, and requires a cAMP responsive element and YY1 binding element to be located in close proximity within *c-fos* promoter [43]. Similarly, the zinc-finger protein Gli2 acts cooperatively with CREB/ATF in the activation of a viral gene, HTLV-1, requiring a combination where the Gli2 binding motif and a cAMP responsive element are in close proximity [44]. Based on these examples of the interaction between CREB/ATF and zinc-finger proteins, we examined the physical interaction between Atf1 and Rst2 under glucose starvation stress, however, no such interaction was detected. This is likely because the interaction is transient/weak and occurs locally at a low frequency. Since weak interactions within Atf1/Php5, Atf1/Php3, Atf1/Php5, and Atf1/Rst2 have been detected in the recently published TF interactome Atlas [45] (https://data.fmi.ch/TFexplorer/pombe.html#), it is possible that these factors reciprocally stabilize each other when in close proximity in the fission yeast genome. Further study is needed to clarify the precise molecular basis underlying such TFs’ self-stabilization mechanisms.

We found that *tup11∆/tup12∆* cells exhibit increased tolerance to heat stress (Fig. 7), suggesting that partial relaxation of Tup11/12-mediated repression may facilitate the induction of heat-responsive genes while Php5-dependent regulation remains intact. Further studies will be required to elucidate the mechanism underlaying the increased heat tolerance of *tup11∆/tup12∆* cells. In contrast, the loss of Tup11/12-mediated gene repression in *php5∆* cells exacerbates heat sensitivity (Fig. 7), highlighting the importance of the dual mechanism of Tup11/12-mediated TF destabilization and Php5-mediated TF stabilization in maintaining transcriptional fidelity and ensuring robust adaptation to a wide range of environmental stresses.

We uncovered a critical role of Tup11/12 and Php5 in enabling fission yeast cells to adapt to external stresses. Tup11/12 is conserved in eukaryotes, corresponding to Groucho in flies and TLE in humans, and plays critical roles in transcriptional regulation, signal transduction, and development [46–48]. Similarly, the role of CBF/NY-F type TFs in the transcriptional regulation is conserved in eukaryotes. Thus, the counteractive regulatory system by Tup11/12 and Php5 might also be widely conserved. Further studies on these regulations in higher eukaryotes might contribute to understanding complex gene expression regulation, and its role in development and disease.

## Supplementary Material

gkag847_Supplemental_Files

## Data Availability

All data are in the manuscript and/or supporting information files. All raw images used in this study are shown in Supplementary Fig. S6 and raw data gained in this study are shown in S7_raw Data. Raw NGS data was deposited at Sequence Read Archive (SRA) under the BioProject accession PRJNA1399038.
