# Supplementary material for: Dual regulation of transcription factor binding by tup11/12-mediated destabilization and DNA loop-mediated stabilization ensures stress-specific gene activation in fission yeast
Source: Nucleic Acids Res. 2026 Sep 7;54(16):gkag847. doi: 10.1093/nar/gkag847 (PMC13546765; doi:10.1093/nar/gkag847)
Supplement: Supplementary file 1 [file gkag847_supplemental_files.zip › Supplementary Information_Tsuruta et al.pdf]

Supplementary Information

Dual Regulation of Transcription Factor Binding by Tup11/12-Mediated Destabilization and DNA Loop-Mediated Stabilization Ensures Stress-Specific Gene Activation in Fission Yeast

Yusuke Tsuruta, Hirotaka Inoue, Kenta Hagiwara, Charles S. Hoffman, and Kouji Hirota

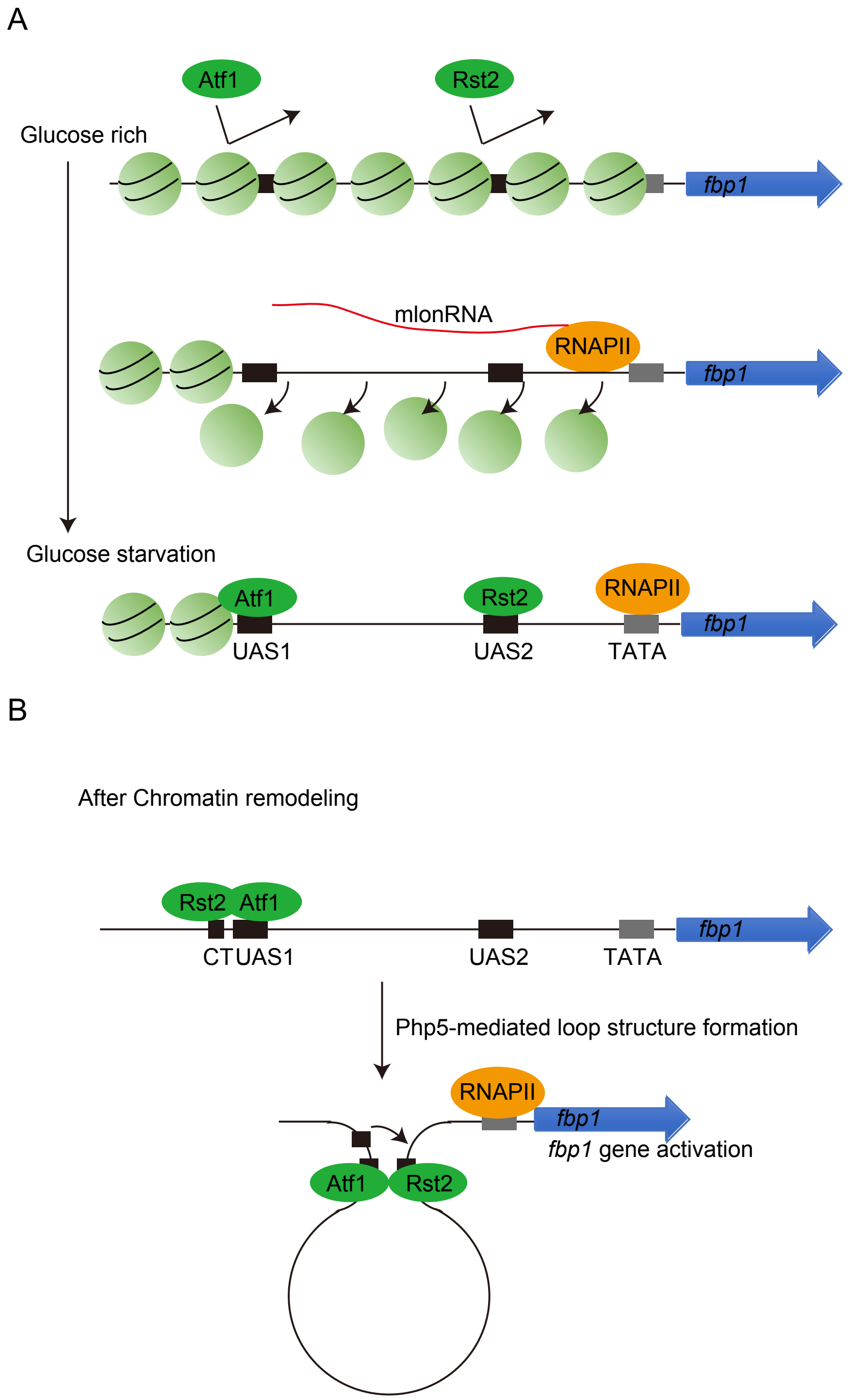

Supplementary Fig. S1 Schematic drawing of the *fbp1* gene induction after glucose starvation stress

(A) UAS1 and UAS2 located in the region upstream from *fbp1* are required for *fbp1* induction. In the presence of glucose, a closed chromatin configuration around these sites represses binding of

transcription factors (TFs). Upon glucose starvation, transcription of a series of ncRNAs called mlonRNAs is induced upstream of *fbp1*, leading to chromatin remodeling and the formation of an open chromatin structure. Atf1 is activated by SAPK under glucose starvation, while Rst2 is repressed by PKA under glucose rich condition. As a result, both of these transcription factors are activated under glucose-deprived conditions. Then, Atf1 and Rst2 become capable of binding to these sites, inducing massive transcriptional activation of the *fbp1* gene. (B) Atf1 and Rst2 first bind near UAS1 and reciprocally stabilize each other and overcome Tup11/12 mediated repression of TF binding. Subsequently, Php5 mediates a loop structure to place UAS1 and UAS2 into proximity, and then Rst2 is delivered from the UAS1 upstream region to UAS2, thereby activating *fbp1* gene expression.

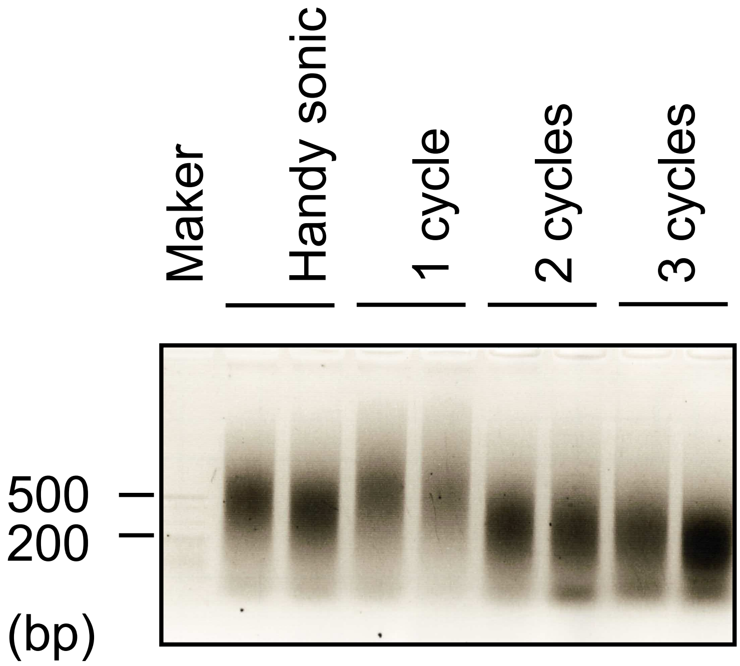

Supplementary Fig. S2 DNA shearing conditions for the ChIP experiment

We compared the degree of DNA shearing under each set of conditions. We compared the conditions used previously (ultrasonic treatment using the handy sonic (TOMY, Tokyo Japan) for 30 sec 6 times) with those used in this study using the Picoruptor (Diagenode, Seraing, Belgium). Under the preset conditions (30 sec in ultra-low-power mode, followed by 30 sec of cooling at 4°C), the ultrasonic treatment cycle was repeated between one and three times. The results showed that a single ultrasonic treatment cycle produced fragments of approximately 500 bp, which was similar to the previous conditions. As three cycles of ultrasonic treatment produced fragments of approximately 200 bp, this condition was adopted.

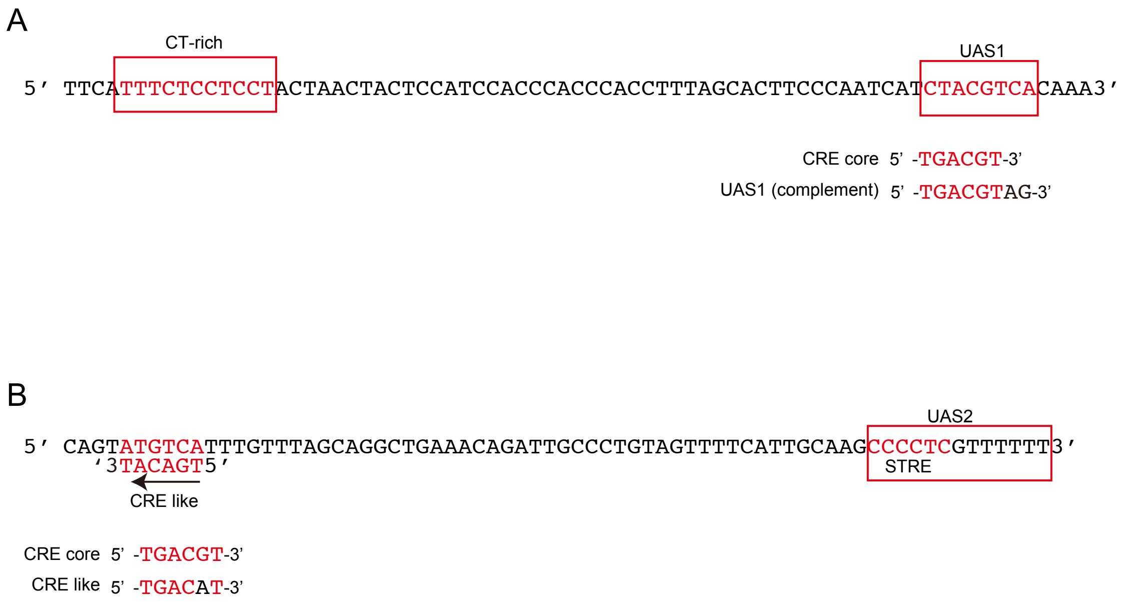

Supplementary Fig. S3 Schematic drawing of the upstream neighbor region of UAS1 and UAS2 in the *fbp1* gene

(A) CT-rich sequence is located 46 bp upstream from UAS1. (B) CRE-like sequence with a 1 bp mismatch in CRE core 6 bases is located 47 bp upstream of UAS2.

fbp1 gene promoter

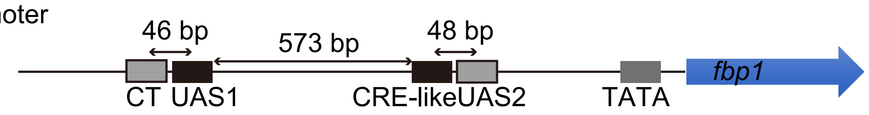

Sequence of each element

CT	5′-CTCCTCCT-3′
UAS1	5′-CTACGTCA-3′ (complementary 5′-TGACGTAG-3′)
CRE-like	5′-ATGTCA-3′ (complementary 5′-TGACAT-3′)
UAS2	5′-CCCCTCGTTTTT-3′

ght3 gene promoter

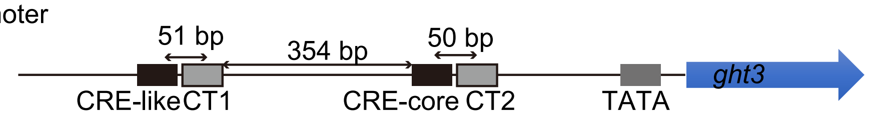

Sequence of each element

CRE-like	5′-ACATCA-3′ (complementary 5′-TGATGT-3′)
CT1	5′-GAGGGG-3′ (complementary 5′-CCCCTC-3′)
CRE-core	5′-ACGTCA-3′ (complementary 5′-TGACGT-3′)
CT2	5′-CTCTTCCTTT-3′

Supplementary Fig. S4 The promoter region of the *ght3* gene possesses a structure like that of the *fbp1* gene promoter region

The promoter regions of *fbp1* (upper) and *ght3* (lower) are presented. Black and gray boxes represent CRE (or CRE-like) and STRE or (CT-rich STRE like) sequences, respectively.

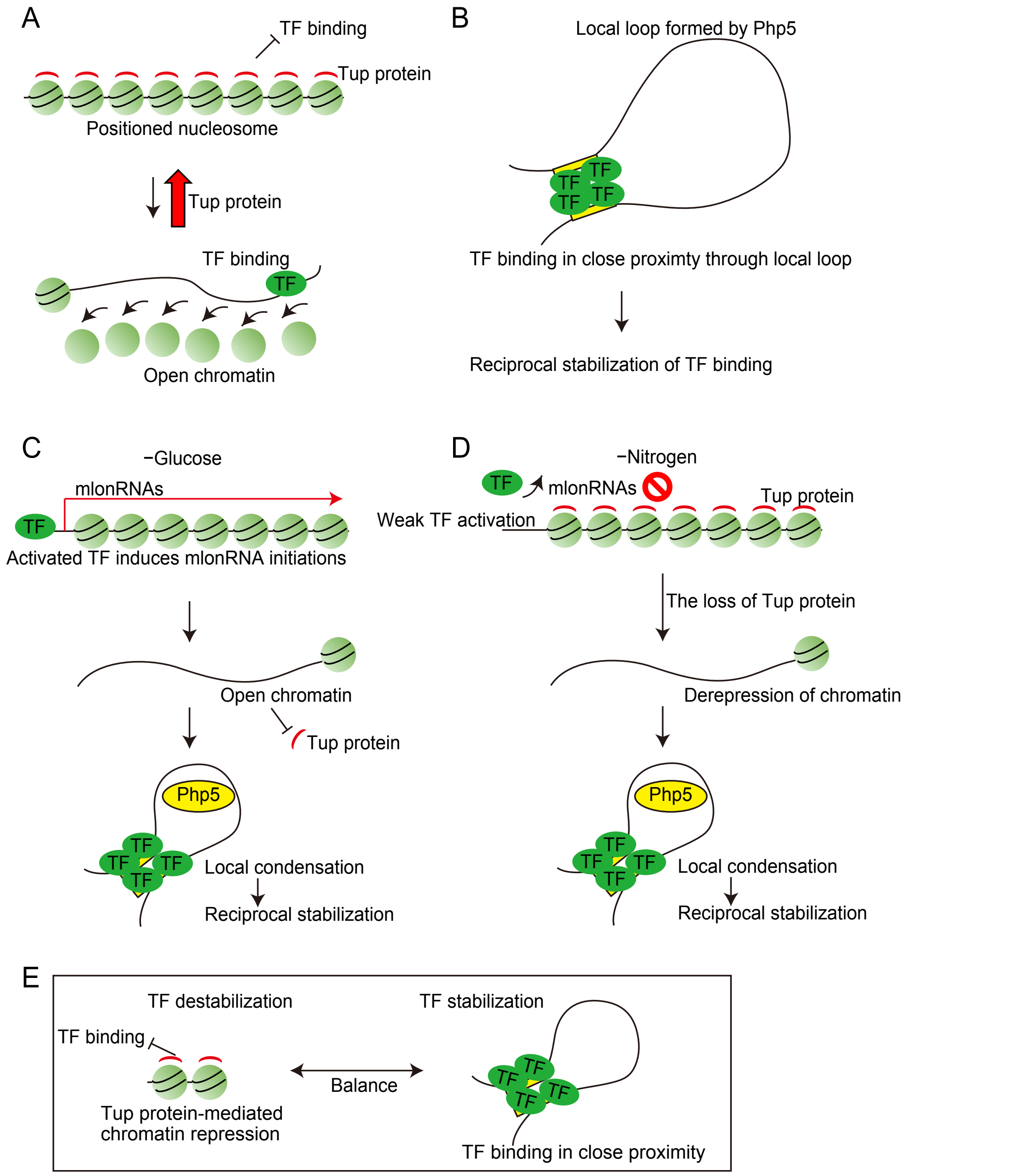

Supplementary Fig. S5 A model illustrating how genes are specifically regulated under various stress conditions

Increasing evidence suggests that there are two regulatory layers: Tup protein-mediated destabilization of TFs trough repressive chromatin (A), and multivalent TF interaction in close proximity stabilizes TF binding (B). (C, D) Mechanisms of TF binding in wild-type and *Tup*ΔΔ cells under glucose starvation and nitrogen starvation conditions. (E) The balance between TF destabilization by Tup protein and TF stabilization by local condensate-like TF reciprocal interaction governs stress-specific responses.

Fig. 1

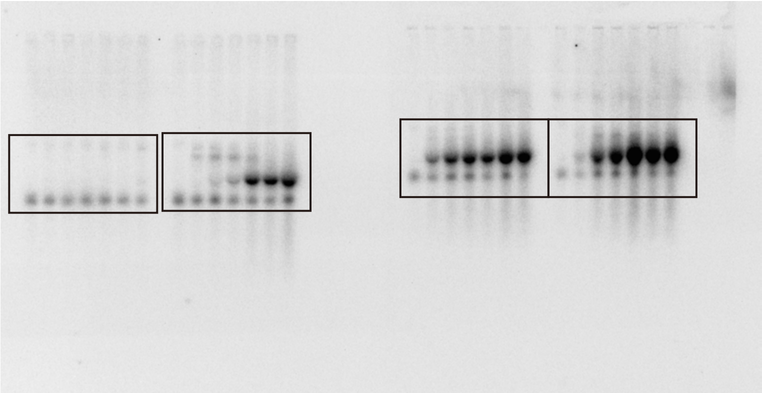

Fig. 2A

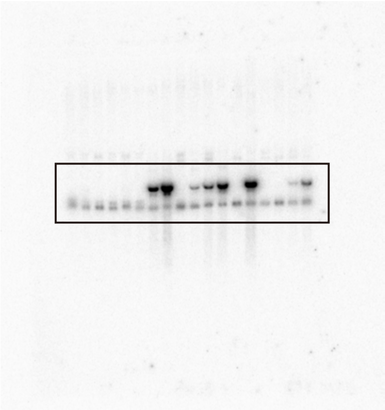

Fig. 4B

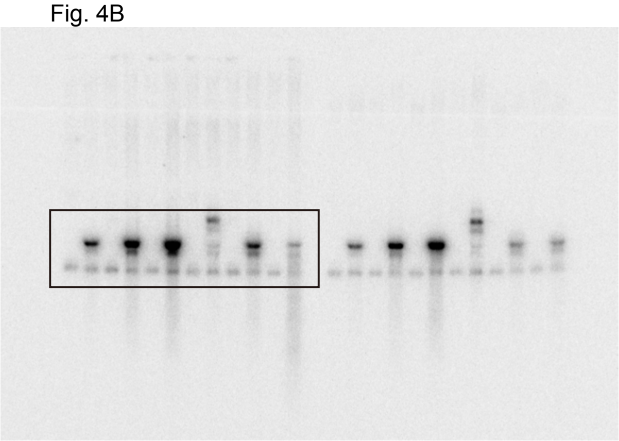

Fig. 3A

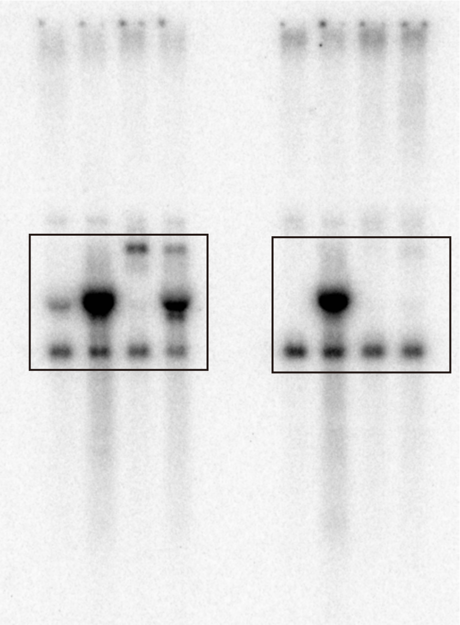

Fig. 4C

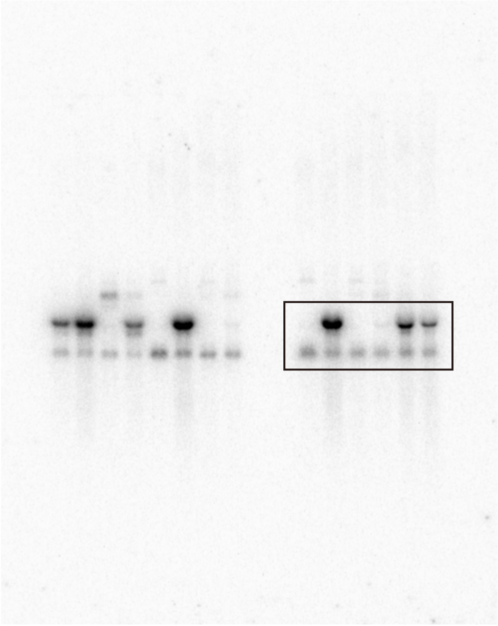

Fig. 5

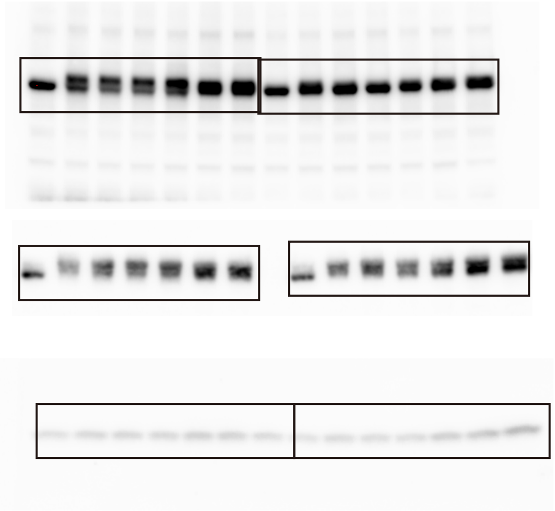

Fig. 6B

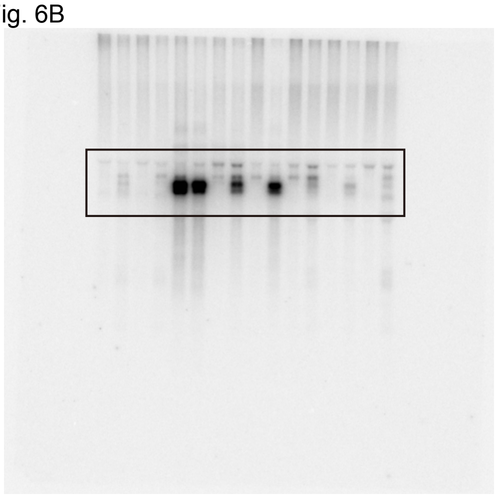

Fig. 6C

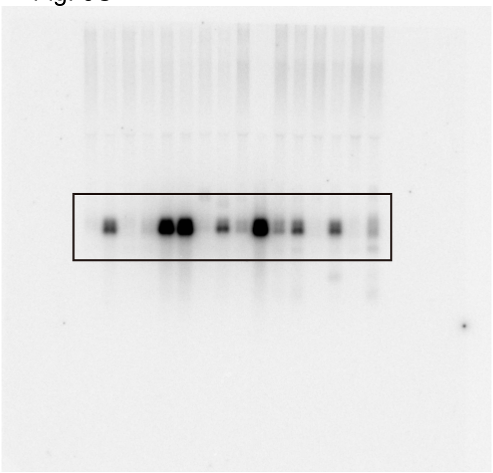

Supplementary Fig. S6 Uncropped raw images used in this study
